# Emerging Biosensing Technologies towards Early Sepsis Diagnosis and Management

**DOI:** 10.3390/bios12100894

**Published:** 2022-10-18

**Authors:** Andrea Bonini, Angela Gilda Carota, Noemi Poma, Federico Maria Vivaldi, Denise Biagini, Daria Bottai, Alessio Lenzi, Arianna Tavanti, Fabio Di Francesco, Tommaso Lomonaco

**Affiliations:** 1 Department of Chemistry and Industrial Chemistry, University of Pisa, Via G. Moruzzi 13, 56124 Pisa, Italy; 2 Department of Biology, University of Pisa, Via San Zeno 35-39, 56100 Pisa, Italy

**Keywords:** sepsis, electrochemical biosensors, optical biosensors, diagnostic, sepsis biomarkers

## Abstract

Sepsis is defined as a systemic inflammatory dysfunction strictly associated with infectious diseases, which represents an important health issue whose incidence is continuously increasing worldwide. Nowadays, sepsis is considered as one of the main causes of death that mainly affects critically ill patients in clinical settings, with a higher prevalence in low-income countries. Currently, sepsis management still represents an important challenge, since the use of traditional techniques for the diagnosis does not provide a rapid response, which is crucial for an effective infection management. Biosensing systems represent a valid alternative due to their characteristics such as low cost, portability, low response time, ease of use and suitability for point of care/need applications. This review provides an overview of the infectious agents associated with the development of sepsis and the host biomarkers suitable for diagnosis and prognosis. Special focus is given to the new emerging biosensing technologies using electrochemical and optical transduction techniques for sepsis diagnosis and management.

## 1. Introduction

**Figure 1:**
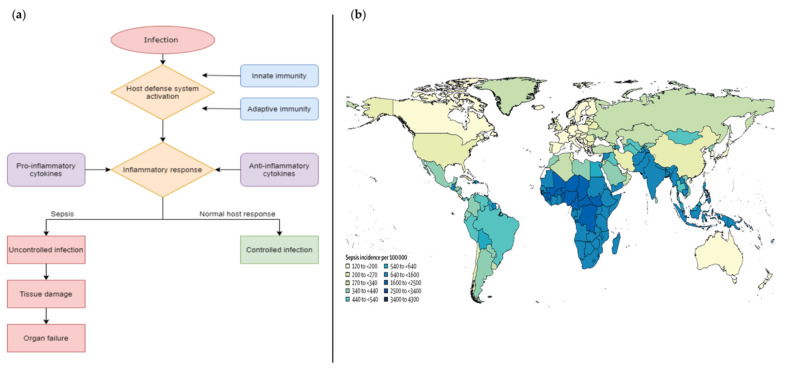
(**a**) comparison of the schematic flow between sepsis and normal host response to an infection; (**b**) geographical incidence of sepsis worldwide [9]. Copyright © 1969, Elsevier.

According to the European Society of Intensive Care Medicine and the Society of Critical Care Medicine (2016 SCCM/ESICM task force), sepsis is defined as “a life-threatening organ dysfunction caused by a dysregulated host response to infection”. This definition highlights the importance of the non-homeostatic host response to infection, the significantly higher lethality than straightforward infection and the need for urgent diagnosis [1]. Sepsis is a “time-dependent” pathology whose clinical outcome depends on the timing of the diagnosis and the effectiveness of clinical management from the very first hour [2]. In fact, if not recognized early and managed promptly, this condition can lead to septic shock, multiple organ failure and death [3,4]. Normally, the sepsis state is the result of the complex host defense response against the invasion from external pathogens such as bacteria and fungi (Figure 1a). After the pathogen invasion, the host defense system is activated to prevent the spreading and multiplication of foreign organisms inside the body which is followed by an inflammatory response, regulated by pro- and anti-inflammatory cytokines [5].

Cytokine signals trigger the cells of the immune system, such as macrophages or neutrophils that rush to the infection site to eliminate the pathogen. This process involves several mechanisms and molecules, including acute phase proteins with several roles and the ultimate objective of controlling the infection.

However, the immune system dysregulated response leads to a situation where severe coagulation provokes microvascular thrombosis and organ dysfunction, which can potentially lead to chronic critical illness or death [6].

Currently, sepsis management is one of the main challenges in clinical settings, especially in the management of critically ill patients. Indeed, it is estimated that each year more than 30 million cases are registered worldwide, with a 9–13% incidence increase each year and a mortality rate of 33–35% [7]. This pathology mainly affects adults aged 65 years or more; people with chronic medical conditions (such as diabetes, lung disease, cancer, and kidney disease), people with weakened immune system and children up to one year of age [8]. In addition, the incidence of sepsis varies by geographical area (Figure 1b) and substantially across regions. The epidemiological trends show the highest burden in sub-Saharan Africa, Oceania, south Asia, east Asia, and southeast Asia, where cases often exceed 600 per 100,000 inhabitants [9], while the prevalence in European countries is about 90 cases per 100,000 inhabitants, leading to 1.4 million estimated cases per year and a mortality rate that fluctuates, depending on the area, between 20 and 40%.

Although the medical guidelines to prevent the infections that can lead to sepsis are continuously updated, sepsis cases are drastically increasing, mainly in low-income countries [10]. In this context, the importance of a rapid and easy diagnosis by means of point of care devices plays a crucial role in this field. Unfortunately, the traditional laboratory techniques are not suitable to respond to this issue, since they require a multi-step analysis, expensive instrumentation, trained personnel, and equipped laboratories. To cope with this need, in recent years lots of efforts have been spent in the development of new biosensing technologies [11,12,13].

Indeed, the characteristics of biosensor devices, such as the speed of response, the portability and the ease of use can effectively improve the diagnosis by helping the physician in the medical decision-making, which would reduce the time and cost of diagnosis. In addition, the routine uses of such point of care devices in the clinical environment close to close to the ward could increase the probability of a patient’s survival.

In this review, the new emerging biosensing technologies for sepsis diagnosis and management are presented. First, an overview of the pathogens associated to the development of sepsis as well as the potential host biomarkers suitable for the diagnosis and prognosis will be provided, followed by a brief description of the traditional laboratory techniques. Finally, special focus will be given to the recent advancement in the development of biosensing technologies, and some examples will be presented.

## 2. Pathogens, Biomarkers, and Conventional Diagnostic Techniques

Nowadays, due to the complex and dysregulated host response, where different mechanisms are involved, there is not an unequivocal biomarker for sepsis identification [14,15]. Indeed, the detection of a single biomarker has low clinical significance, and only the identification of several biomarkers and physiological parameters provides the physician with a framework of useful diagnostic elements for evaluating the patient’s condition.

From the clinicians’ routine point of view, in the case of suspicion of sepsis, a quick evaluation of wide physiological parameters is applied. This diagnostic methodology is called “qSOFA” (quick sequential organ failure assessment score), which is a score system based on respiratory, neurological, and hemodynamic variables. This method shows a better performance in specificity than other similar methos, such as “SIRS” (systemic inflammatory response syndrome) and “NEWS” (national early warning score), but worse results in sensitivity [16].

At the same time, the microbiological analyses should be performed immediately to identify the pathogen and to rapidly orient the antibiotic administration [10]. Meanwhile, several biomarkers are analyzed and evaluated to acquire additional information about the infection (e.g., to distinguish between viral and bacterial infection) and determine the patient’s condition [14,17].

### 2.1. Pathogens

Not all pathogens can cause sepsis, as bacteria need specific features to overcome the defense barriers, survive, proliferate, and disseminate in the human body. Most of such pathogens are facultative aerobic or anaerobic microorganisms with effective defense systems against oxidative stress, such as the production of superoxide dismutase (SOD), catalase and glutathione peroxidases.

Different virulence factors have been identified, such as exotoxins for gram-positive and endotoxins (e.g., lipopolysaccharides and LPS) for gram-negative bacteria. Although gram-positive bacteria lack endotoxins, they invade host tissues more easily due to the presence of exposed peptidoglycans and a range of other toxic secreted products [7]. A list of sepsis-causing bacteria is reported in Table 1.

### 2.2. Biomarkers

Hundreds of potential biomarkers have been proposed for the diagnosis and prognosis of septic patients. Generally, the main attributes of successful and effective biomarkers are high sensitivity, specificity, possibility of bed-side monitoring, and financial accessibility. However, these criteria are only met by a few parameters that can be potentially used in the clinical practice for a timely and reliable diagnosis. An important feature for an effective biomarker would be the capability to discriminate between inflammations of infectious or non-infectious origin, as this aspect significantly influences the success of therapies [14]. The main sepsis biomarkers and their characteristics are summarized below.

#### 2.2.1. C-Reactive Protein (CRP)

CRP is a protein released in plasma by the liver whose concentration rises 24–38 h after inflammation develops. Physiologically, it binds to the lysophosphatidylcholine expressed on the surface of dead or dying cells (and some types of bacteria) to activate the complement system and stimulate the opsonization and phagocytosis [19]. The CRP concentration in healthy subjects is lower than 5 mg/L, and its level is diagnostically used to differentiate between viral and bacterial infections. CRP is not a specific biomarker for inflammation associated infections since its concentration is also elevated in many other pathological conditions [14].

#### 2.2.2. Procalcitonin (PCT)

PCT is a calcitonin precursor protein that, in physiological conditions, is secreted by the C cells of the thyroid gland and stored in the Golgi apparatus. Consequently, plasma PCT is normally at trace levels but it increases during the sepsis state due to its production by macrophages and monocytic cells of different organs, especially of the liver. Procalcitonin acts as chemokine, modulating the induction of anti-inflammatory cytokines and inducing the production of nitric oxide synthase [14]. During bacterial infections in adults, PCT serum starts increasing 4 h after the onset of a systemic infection and peaks between 8 and 24 h, with an estimated half-life of approximately 22–29 h [20]. In viral infections, only a minimum increase in PCT concentration is observed [14].

#### 2.2.3. Lipopolysaccharide Binding Protein (LPB)

During the acute phase of inflammatory response, LBP is produced by the liver to help lipid A or bacterial lipopolysaccharide to bind a cluster of proteins on monocytes and macrophages [21,22]. Under physiological conditions, serum concentrations fluctuate between 5 and 15 μg/mL [14], while during sepsis average values become 30–40 μg/mL within 24 h [21]. A meta-analysis performed by Chen K-F et al. showed a weak sensitivity and specificity for sepsis [21], even if its prognostic importance has been demonstrated [14].

#### 2.2.4. D-Dimer (DD)

Circulating D-dimer, a degradation product of cross-linked fibrin, is widely used as a fibrin-related marker for diagnostic and prognostic purposes, but its prognostic value in sepsis, either alone or in combination with other biomarkers, needs further validation. Since DD formation depends on coagulation and fibrinolysis, it may yield negative results in conditions associated with pronounced fibrinolytic inhibition such as sepsis. A recent study showed that the correction of DD for thrombin and plasmin generation may represent a new prognostic marker in septic patients [23].

#### 2.2.5. Interleukins (ILs) and Other Cytokines

ILs are a group of cytokines, i.e., proteins acting as signal molecules both between cells of the immune system and between these cells and different organs and tissues. In particular, IL-6, IL-8, and IL-10 are used to diagnose sepsis, to assess the level of inflammatory response and to help the prognosis. IL-6 is a proinflammatory cytokine produced by cells such as monocytes, fibroblasts, endothelial cells, keratinocytes, T-lymphocytes, and tumor cells. It is released into the bloodstream 4–6 h after the increase in LPS, whereas IL-6 concentration decreases after 24–48 h of the presence of viable bacteria.

IL-8 is the main chemokine produced by macrophages and endothelial cells. It is considered a good predictive marker of sepsis in pediatric patients, but not for adults [14].

IL-10 is an anti-inflammatory cytokine produced by macrophages, monocytes, neutrophils, T and B lymphocytes, and mesangial cells. High levels of both IL-6 and IL-10 are related to mortality of septic patients [14].

Other cytokines are involved in sepsis and septic shock, such as TRAIL and IP-10, whose levels are significantly increased in septic patients [24]. TRAIL is a potent inducer of apoptosis, whose levels are associated with in-hospital mortality, organ dysfunction, and septic shock [25]. All these cytokines allow for a quantitative assessment of the severity of sepsis.

#### 2.2.6. Surface Markers of Circulating Leukocytes

Several studies point out the importance of surface markers of circulating leukocytes, such as Cluster of Differentiation 64 (CD64), for the diagnosis of sepsis in neonatal and adult patients [26,27,28].

CD64 is a type of integral membrane protein that binds monomeric IgG-type antibodies with high affinity [28,29]. Currently, there is a standard test called Trillium Diagnostic’s Leuko64 for the determination of the expression of CD64 on neutrophils, which represents a positive step in the sepsis mosaic [15].

#### 2.2.7. Fibronectin (FN)

FN is a high-molecular weight glycoprotein that plays an important role in cell adhesion and migration, anti-infection, hemostasis, injury repair and maintenance of microvascular integrity.

In typical physiological conditions, the FN plasma concentration is about 200–600 μg/mL (0.4–1.2 μM), but this value decreases in patients with severe infection and is closely related to the severity of sepsis [30].

#### 2.2.8. Lactate Dehydrogenase (LDH)

LDH is an enzyme catalyzing the conversion of pyruvate to lactate by reducing NAD^+^ to NADH [31]. Increased LDH levels in serum indicate tissue injury, hypoxia, necrosis, malignancies, hemolysis, but it is also associated with mortality in septic patients [32,33]. Erez et al. reported LDH as an independent parameter for predicting the mortality of any hospitalized patient [34]. According to Zein et al., LDH levels that do not stabilize within the first 48 h of inflammation are a significant indicator of mortality in patients with severe sepsis [35].

#### 2.2.9. MicroRNAs (miRNAs)

MiRNAs are small (20–24 nucleotides) RNA molecules that regulate gene expression. miRNA genes are estimated to represent only about 1% of the human genome but are thought to regulate up to 60% of all protein-coding genes. MicroRNAs belong to complex networks regulating gene expression in physiological and pathophysiological processes. The disruption of highly regulated mechanisms such as development aging, cell death may be associated to the aberrant miRNA expression, interestingly this abnormal expression can also be identified in diseases associated with infection and sepsis. The expression of IL-6, TNFα and other sepsis biomarkers is in fact regulated by miRNAs. Consequently, circulating miRNAs could be used as diagnostic biomarkers of sepsis, providing rapid information about infections compared to the traditional microbiological methods. Nevertheless, further studies should be performed to improve the understanding of miRNA concentrations in septic patients [36].

### 2.3. Traditional Laboratory Techniques

**Figure 2:**
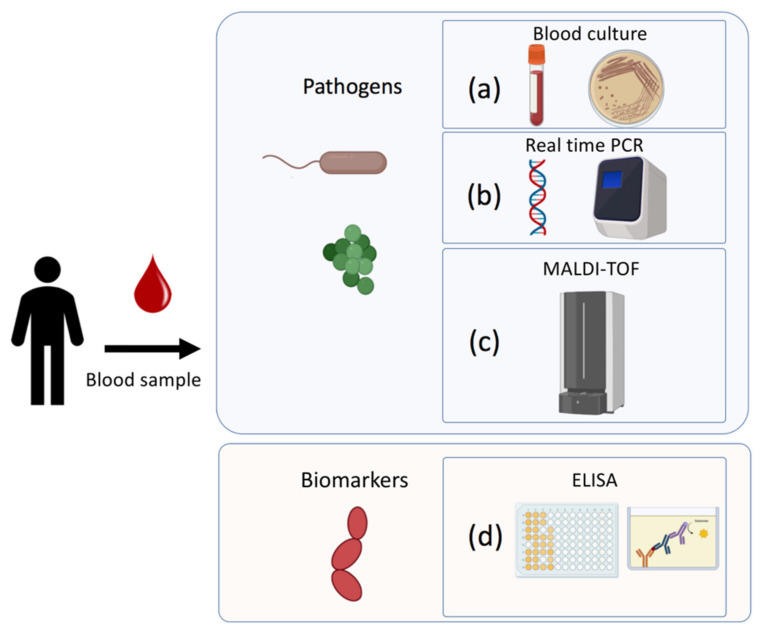
Traditional techniques to detect pathogens and biomarkers associated to sepsis: (**a**) Blood culture-based approach for microorganism identification; (**b**) DNA/RNA amplification-based technique (RT-PCR); (**c**) Matrix-Assisted Laser Desorption/Ionization Time-of-Flight Mass Spectrometry; (**d**) Immunoenzymatically serological assay for biomarkers detection.

A rapid and effective diagnosis of sepsis is fundamental in clinical settings, since each hour of delay in the identification and administration of antimicrobial therapy, drastically increases the mortality of the patient [14]. Currently, the traditional techniques used are classified in culture-based approaches, molecular techniques, and serological analysis. In the following paragraphs, the main techniques used to diagnose the infection and its associated pathogens and biomarkers will be discussed (Figure 2).

#### 2.3.1. Blood Cultures (BCs)

The blood culture-based approaches are the historical and traditional ones, encompassing several laboratory identification methods (e.g., gram staining, biochemical tests, etc.), that provide information on the bacterial species and are usually flanked by antibiotic susceptibility testing [37]. BCs are usually performed by automated instruments/tests (BacT/ALERT 3D bioMérieux, Marcy l’Etoile, France, or BACTEC Becton Dickinson B. V., Breda, Netherlands) that continuously monitor bacterial growth [38]. However, the long time needed for the definitive identification of the organism responsible for the bacteremia and its antibiotic susceptibility testing (usually more than 1 day) delays the administration of proper antibiotic and supportive treatments [39]. In addition to this issue, some studies reported that BCs tests are not suitable for the neonatal patient, because they require a high sample volume (at least 5 mL), and these techniques can fail in the identification of slow-growing pathogens as is case of previous antimicrobial treatment [40,41]. For this reason, the usefulness of blood cultures sampling at admission in emergency departments has been recently questioned [42], and new molecular techniques are becoming the new routine tests.

#### 2.3.2. Molecular Methods

Recently developed molecular diagnostic techniques are able to rapidly provide information on the infecting pathogen with high sensitivity and level of confidence [37]. The molecular methods generally employed can be classified in nucleic acid amplification-based (based on polymerase chain reaction (PCR)) and in protein-based techniques such as Matrix-assisted laser desorption/ionization time-of-flight mass spectrometry (MALDI-TOF).

##### Nucleic Acid Amplification-Based Techniques

Amplification methods allow for the enhancement of the detection signal in complex samples and the identification of target sequences associated to a bacterial species, or antibiotic resistance genes. Real-time quantitative PCR (RT-qPCR) amplifies and simultaneously detects the presence of nucleic acids in a sample. The technique is easy to perform for trained staff and is characterized by high specificity and sensitivity. For this reason, it is considered a suitable alternative to culture-based methods in a clinical microbiology laboratory. RT-qPCR relies on the use of specific oligonucleotide primers to amplify a DNA substrate, a polymerase, an intercalating fluorescence probe and precise thermal step cycles. In general, it only takes two hours to overcome the amplicon threshold needed to obtain a recordable signal. Two major drawbacks of these techniques are the risk of contamination, making a certain dose of expertise necessary, and the presence of false positives, due to amplification of non-target genes for the aspecific annealing of primers [38]. Importantly, this technique can be performed directly on whole blood samples (usually 500 µL of volume) allowing for a rapid identification of the pathogen. The direct diagnosis from whole blood samples circumvents the drawbacks of blood culturing approaches, in particular for slow-growing bacteria or non-culturable microorganisms, and in cases when the patient has already received antimicrobials [43].

From a commercial point of view, multiplex PCR assays on whole blood are already available. SeptiFAST (Roche Diagnostics, Mannheim, Germany) was the first commercial multiplex assay for the detection of pathogens directly from blood and consequently it is mostly studied. It is important to mention that molecular diagnostic tests are expensive if compared to culture-based and phenotypic methods but are generally less laborious and faster. In a cost-effectiveness study, SeptiFAST was assessed to provide a significant economic saving [44].

##### Matrix-Assisted Laser Desorption/Ionization Time-of-Flight Mass Spectrometry (MALDI-TOF)

MALDI-TOF is now a routine method in several laboratories as it allows for easier and faster diagnosis of human pathogens than conventional phenotypic and molecular identification methods, with reliability and cost-effectiveness [45]. This technique allows for the identification of species according to their unique proteomic profile, which is obtained from post-culture samples [46,47]. In addition, it has been demonstrated that it is able to rapidly detect antibiotic resistant strains as shown by Kempf et al., with the spectrum of susceptible strain showing a peak matching with the drug’s spectrum, while resistant strains show different peaks corresponding to degradation products of the drug [48].

Although MALDI-TOF is a promising technique for the identification of bacteria and for a rapid evaluation of antimicrobial resistance, a major drawback is that the reference database must be regularly extended to allow for matching of uncommon strains or species. MALDI-TOF MS is characterized with a high sensitivity and specificity, and the cost of the reagents is low, but many laboratories cannot afford the initial investment for the instrument. Moreover, the technique is suitable for high-throughput analyses reducing costs [48,49].

#### 2.3.3. Serological Methods

The diagnosis and management of sepsis relies on heterogeneous information including biomarker levels, which are usually assessed through immunoassay. These include a plethora of qualitative or quantitative analytical techniques for the detection and measurement of many clinically relevant analytes.

The immunochemical techniques rely on the ability of antibodies to specifically bind antigens such as proteins, carbohydrates, and other molecules [50]. The commonly used immunochemical assay is the enzyme-linked immunosorbent assay (ELISA) in its ‘’sandwich’’ strategy [51]. In this assay an aliquot of sample containing the analyte is added into a polystyrene microtiter plate where a known amount of antigen-specific antibody is bound. After washing, an enzyme-labeled antibody is added, forming a “sandwich complex” triple layer of Antibody-Antigen-Antibody-enzyme. After washing away the unbound antibody, the enzyme substrate is added and an amount of colored product forms that is proportional to the amount of analyte in the sample [50].

Despite its clear advantages, ELISA has some limitations, such as the laborious procedure and the insufficient level of sensitivity towards certain biomolecules [52]. Lastly, to detect a given antibody or antigen, a known reciprocal antigen or antibody must be generated [53].

## 3. Biosensor as an Alternative Device for Sepsis

A biosensor is an analytical device that converts chemical/biochemical information into a useful analytical signal [54,55,56]. It is always composed of two basic elements: a bioreceptor, a selective and specific biological recognition element such as enzyme(s), DNA, antibodies among others; and a transducer, which converts the receptor-analyte interaction into an analytical (i.e., optical or electrical) signal whose intensity is directly or inversely proportional to the analyte concentration [57].

Biosensors can be classified based on their applications, and more in general the biosensor should fulfil the following characteristics such as: low cost, portability, low response time, ease of use and suitability for point of care/need applications [58]. Even though many different types of biosensors have been described, in this review we have examined the most recent emerging electrochemical and optical biosensors developed for the early detection of sepsis.

### 3.1. Electrochemical Biosensors

**Figure 3:**
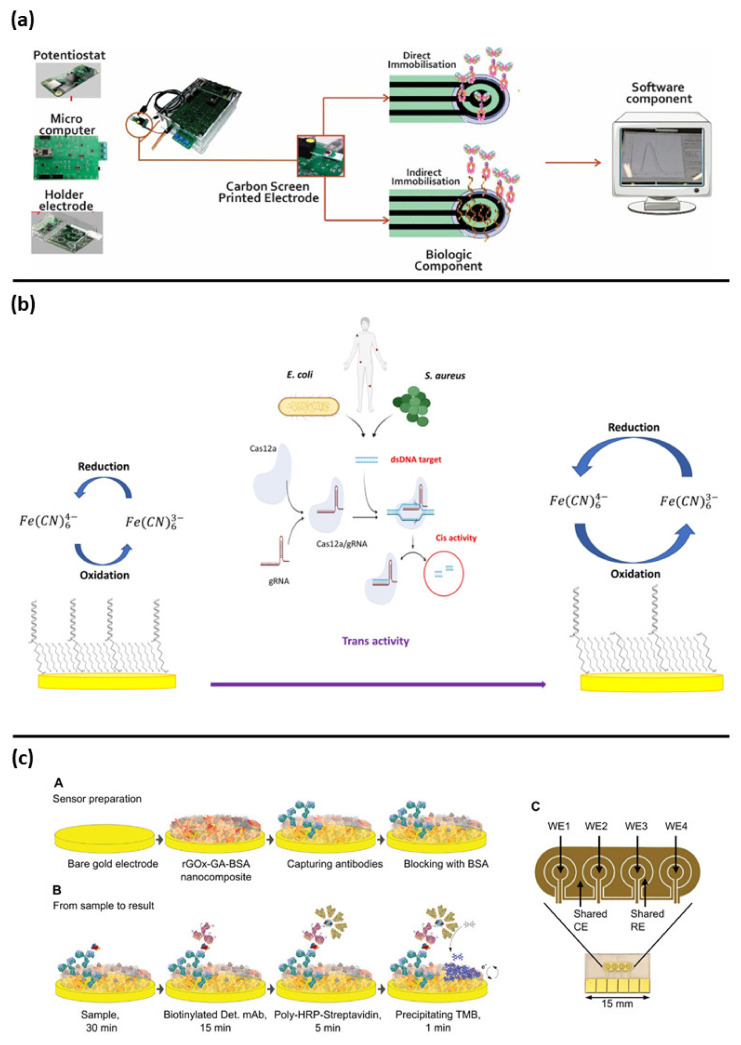
Examples of electrochemical biosensors approaches for sepsis diagnosis: (**a**) representation of all components of point of care biosensor for CRP detection, reprinted from [68] Creative common CC BY 4.0; (**b**) assay schematic of CRISPR/cas12a based biosensor for *E. coli* and *S. aureus* detection, reprinted with permission of [81], copyright 2021 Elsevier B.V.; (**c**) fabrication steps and electrode schematic for PCT and CRP detection, reprinted with permission [73], copyright 2021 Wiley-VCH GmbH.

**Figure 4:**
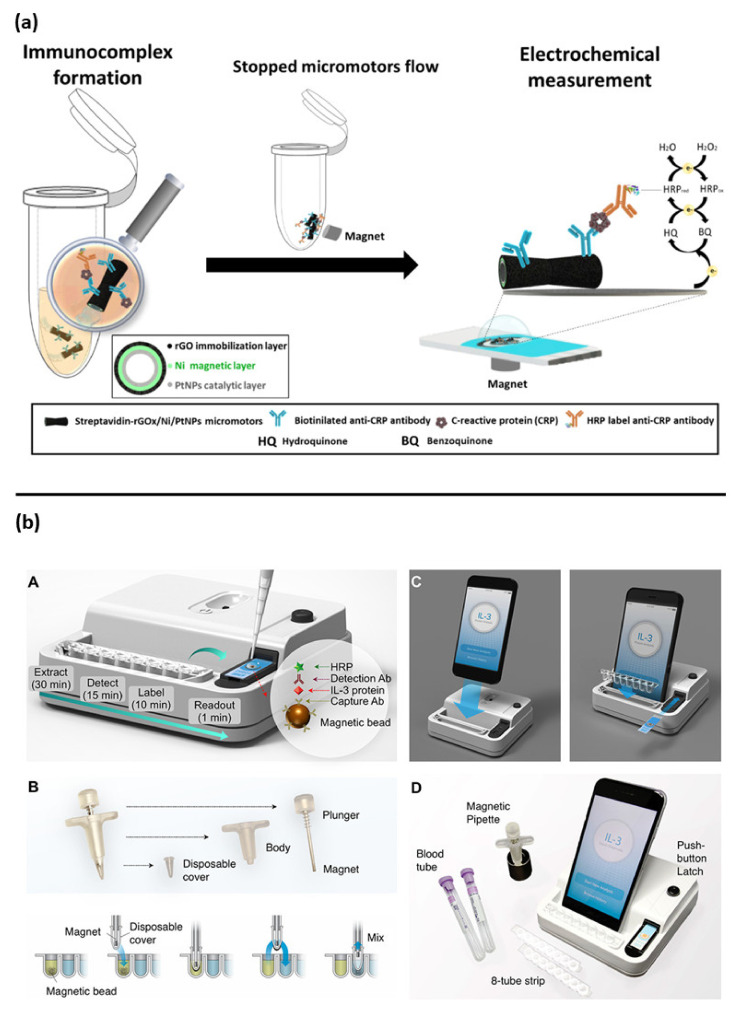
Other examples of electrochemical biosensor approaches for sepsis diagnosis: (**a**) magnetic micromotors-based assay immunoassay for CRP detection, reprinted with permission [67] Copyright 2020 Elsevier B.V; (**b**) assay schematic of point of care integrated sensor for cytokines detection, reprinted with permission of [76] Copyright © 2018, American Chemical Society.

Electrochemical biosensors (Figure 3 and Figure 4) combine the sensitivity and the low response time of electroanalytical methods with the selectivity and specificity of the biological recognition element.

Electrochemical sensing usually requires a working electrode (WE), a reference electrode (RE), and a counter/auxiliary electrode (CE). Reactions are detected only near to the WE surface, which has a key role in determining the detection ability thanks to its dimensions, nano/materials and bio element and modification [59,60]. Electrochemical sensors are generally classified as amperometric, potentiometric, impedimetric and conductometric sensors, according to the electroanalytical technique they use [54,56,61]. Furthermore, these biosensors are suitable for the miniaturization and integration in microfluidic and low-cost point of care devices [62,63,64]. Herein, we report the emerging electrochemical biosensors developed for sepsis diagnosis (Table 2).

#### 3.1.1. Procalcitonin (PCT), C-Reactive Protein (CRP) Detection

Different emerging biosensors have been described in literature to detect PCT and CRP. These systems used a label-free approach for a single analyte to a multi-analyte detection platform, taking the advantage of the latest improvements of the engineering and the nano-(bio)technologies fields.

In 2017, Lim et al. developed a new label-free biosensor for the rapid detection of PCT based on recently discovered PCT-Binding Protein 3 (BP3 peptide) and electrochemical impedance spectroscopy (EIS) as transduction technique [65]. The peptide was immobilized onto a gold electrode and detection was performed in a buffer solution.

Another label-free approach was proposed by Guillem et al., for the CRP detection. The authors showed an interesting low-cost point of care device associated to open-source electronic readout elements (Figure 3a). The device was based on a carbon screen printed electrode functionalized with antibodies. This was able to detect the analyte in a small sample volume (50 µL), both in buffer and in spiked plasma [68].

The use of nanotechnologies was described by Ge and collaborators. They developed a label-free electrochemical immunosensor based on the synergic effect of two different nanostructures the AuPtCu nanodendrites coupled with graphene-wrapped Co nanoparticles encapsulated in 3D N-doped carbon nanobrushes used to modify the electrode surface. These nanostructures have improved both the antibody loading capacity of the electrode and the mass/electron transport catalyzing the H_2_O_2_ reduction, reaching an ultra-low LOD around 0.011 pg/mL in diluted serum samples [69].

Others similar works based on nanomaterials strategies are listed in Table 2 [70,71].

Interesting new approaches for PCT and CRP detection are based on magnetic-assisted workflow improving the sensitivity and selectivity of the biosensor. Águeda Molinero-Fernández et al. (2020) have developed a biosensor with a sandwich immunoassay configuration for the detection of PCT using magnetic beads [66]. Streptavidin coated magnetic beads (MBs) were functionalized with anti PCT antibodies, then disposable screen-printed carbon electrodes (SPE-C, on-drop detection) and electro-kinetically driven microfluidic chips with integrated Au electrodes (EMC-Au) were used. The amperometric measurements demonstrated a lower LOD with the EMC-Au electrode. In a lateral study, the same group also described a magnetic assisted immunoassay for the detection of CRP in neonatal septic patients, in a very small plasma sample volume (<10 µL), using an innovative system based on micromotors (Figure 4a) [67]. In that study, bubble-propelled micromotors converted chemical energy into autonomous propulsion, moving within the sample and binding the analyte. Micromotors consisted of an inner catalytic layer of platinum nanoparticles, which allowed the reaction responsible for propulsion, and an outer layer of reduced graphene oxide (rGO) functionalized with anti-CRP antibodies to bind the analyte. The CRP detection was based on an amperometric sandwich assay by using functionalized micromotors, a secondary antibody conjugated with an enzyme (Horseradish peroxidase: HRP) and a screen-printed carbon electrode. Micromotors offer an important advantage compared to traditional microbeads, since moving the bioreceptor within the sample increases the chances of interaction with the analyte and hence the sensitivity, which is usually limited by an inefficient transport of the analyte towards the bioreceptor.

With the aim to improve the fundamental diagnostic information for medical doctors’ decisions, multianalyte devices able to simultaneously detect PCT and CRP biomarkers have been developed. Ambalika Sanjeev Tanak and et al. have shown the possibility to detect both of PCT and CRP at the same time [72]. Such dual marker biosensing strategy consisted of two gold interdigitated electrodes on a flexible polyimide substrate coated with a thin film of ZnO, functionalized with specific antibodies. PCT and CRP were measured in human serum and whole blood. A very impressive and useful output was provided from Zupančič et al. The authors described an electrochemical immune biosensor able to detect PCT and CRP in buffer solution as well as in serum and whole blood samples collected from clinical patients. The biosensor was able to discriminate between the infected and non-infected groups. These results agreed with a standard ELISA kit and shown the possibility to use this sensor as a POC device. The sensor was based on a gold surface functionalized electrode with a 3D nanocomposite containing crosslinked bovine serum albumin (BSA) doped with conductive reduced graphene oxide nano flakes (rGOx) nanomaterials functionalized with antibodies; this used a sandwich assay approach for the detection of the biomarker of interest (Figure 3c) [73].

#### 3.1.2. Cytokines

Since cytokines are involved in several mechanisms during the host response to infection, they are used as convenient biomarkers for sepsis detection (Section 2.2.5). Among this class of biomarkers, most of the developed biosensors are referred to detect the interleukins (Table 2).

Russell et al. [74] developed a microelectrode for the real time electrochemical detection of IL-6 using a needle shaped silicon substrate bearing eight gold disc electrodes functionalized with an antibody for IL-6. Measurements were carried out by using EIS and DPV techniques. The study demonstrated the possibility to detect IL-6 in clinically relevant samples without the need of complex electrode modifications or labelling steps.

Chen et al. proposed a label-free capacitive immuno- nano-biosensor based on a gold interdigitated electrode modified with longitudinal zeolite and iron oxide-complexed nanocomposite functionalized with antibodies to diagnose IL-3. The biosensor was able to detect IL-3 with a LOD of 3 pg/mL in a spiked human serum.

Specifically, for IL-3 detection, Min at al. have described an interesting magneto electrochemical sensor, integrated with a simple smartphone readout for POC applications (Figure 4b). This assay was able to rapidly detect the IL-3 in (<1 h) with a LOD of <10 pg/mL in human plasma samples [76].

The diagnostic information would definitively improve if more biomarkers could be detected simultaneously. In this sense, similarly to what was described for other biomarkers, methods to detect multiple different interleukins simultaneously have been explored. Ambalika S. Tanak et al. [77] demonstrated a novel multiarray point of care device that directly monitored a panel of five cytokine biomarkers (e.g., IL-6, IL-8, IL-10, TRAIL and IP-10). The device enclosed an array of gold electrodes coated with a nanofilm of semiconductive ZnO, functionalized with specific antibodies. The binding interaction when using plasma samples was registered by using EIS. This device was able to determine different information about patient’s condition. In fact, the combination of pro- and anti-inflammatory markers (e.g., IL-6, IL-8, and IL-10) can reveal the host immune response during the early stages of sepsis, while the detection of TRAIL and IP-10 may provide information about the origin of the infection, since these two biomarkers are differentially expressed in viral and bacterial infections [82]. In 2022, the same research group have tested and validated the previously biosensor developed named Direct Electrochemical Technique Targeting (DETecT) sepsis device with a 124 sepsis patients’ samples. Therefore, they have detected not only the interleukins group but were added the PCT and CRP biomarkers. The data was compared and resulted to agree with the LUMINEX standard method, opening the possibility to use this device to obtain a lot of information for sepsis diagnoses [78].

#### 3.1.3. Pathogens

As reported in Section 2.2, despite the urgent need for a quick pathogen identification, the traditional laboratory diagnostic techniques are rather slow. In response to this issue, a study conducted by Gao et al. (2016) [79] reported a multiplex electrochemical biosensor for the rapid identification of pathogens in blood samples. This biosensor detected the species-specific sequences of the 16S ribosomal RNA of both gram-positive and -negative bacteria such as *S. aureus*, *E. coli*, *P. aeruginosa* and *P. mirabilis*. Gold electrodes were deposited on a plastic substrate, where each chip was composed of 16 electrodes. A sandwich strategy was used to detect the analyte on every gold electrode, and different thiolated oligonucleotide probes (universal and specific ones) were immobilized on the electrode surfaces. After the interaction between capture probe and 16S rRNA, an HRP targeted DNA was added as a secondary detection probe to catalyze the redox reaction of H_2_O_2_, whose current signal was amperometrically registered by a multichannel potentiostat.

Another interesting approach was proposed by Sharma et al. [80] who reported the use of a molecularly imprinted polymer (MIP) as the recognition element to detect a *Klebsiella pneumoniae*. The MIP was synthetized by using polypyrrole (PPy) a conductive polymer and the bacterium served as a template. Difference pulse voltammetry DPV was used as the transduction technique and the sensor was able to detect the bacteria in buffer solution with a LOD of 1.35 CFU/mL.

Based on the new and recently discovered clustered regularly interspaced short palindromic repeats (CRISPR) and associated protein systems (Cas) named CRISPR/Cas system, Bonini et al. developed a label-free electrochemical biosensor for the bacterial DNA detection. The authors shown the possibility to detect *E. coli* and *S. aureus* bacterial DNA from clinical isolates. This biosensor was based on a DNA functionalized gold electrode and took advantage of the programmability of the Cas12a/gRNA enzyme using its primary and collateral activities for the specific bacterial detection and the signal amplification, respectively (Figure 3b) [81,83].

### 3.2. Optical Biosensors

An optical biosensor is a compact analytical device containing a biorecognition sensing element integrated with an optical transducer [84]. An optical transduction can be achieved by measuring the light power absorbed or emitted by a component of the sensing layer at a specific wavelength. Optical methods need a component in the sensing layer that absorbs or emits light: whenever this condition is not fulfilled, optical signaling labels have to be used. An alternative transduction is represented by the optical monitoring of a physical property (e.g., the refractive index) of the sensing layer that varies upon the interaction with the analyte. These transduction techniques do not need an optical label and are denoted as label-free methods [85,86,87,88].

Recent optical biosensors developed for sepsis diagnosis together with their characteristics, grouped by analyte, are shown in (Table 3).

#### 3.2.1. Procalcitonin (PCT), C-Reactive Protein (CRP) Detection and Interleukins

**Figure 5:**
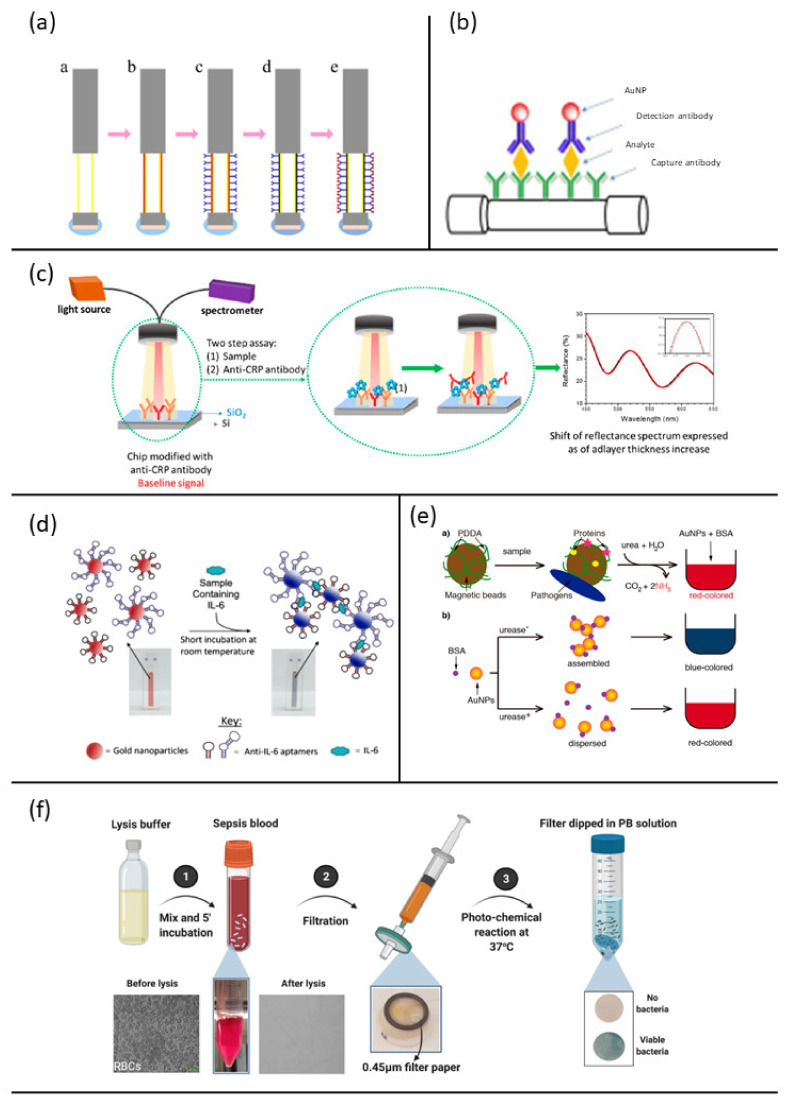
Examples of optical biosensor approaches for sepsis diagnosis: (**a**) steps for fabrication of the fiber optic SPR biosensor fort the CRP detection, reprinted with permission of [89] Creative common CC BY 4.0; (**b**) schematic sandwich assay for PCT detection, reprinted with permission of [90] Copyright 2019 Elsevier B.V.; (**c**) schematic sensing principle for CRP detection, reprinted from [97] Creative common CC BY 4.0; (**d**) schematic of the aptamer-gold nanoparticle-based assay for the detection of mouse IL-6, reprinted from [91] Creative common CC BY 4.0; (**e**) schematic representation of the method for detect urease positive bacteria involved in sepsis, reprinted with permission of [93], Copyright © 2019, American Chemical Society; (**f**) schematic showing the working principle of selective isolation and detection of bacteria from whole blood involved in sepsis, reprinted [95], Creative common CC BY 4.0.

In 2017, Wang et al. [89] developed a label-free biosensor using a fiber optic and surface plasmon resonance (SPR) for the specific detection of CRP. Such biosensor included a multi-mode fiber as the optical waveguide coated with a gold film in which plasmons could be generated. The Au layer of the sensor was functionalized by using dopamine as a crosslinking agent to immobilize the anti-CRP monoclonal antibody used as the selective ligand (Figure 5a). When the analyte bound the antibody, the change in refractive index of the medium through which the plasmonic wave was propagating shifted the reflectance angle. The magnitude of the shift depended on the amount of captured analyte, and the shift could be measured in almost real time [98]. This sensor showed good selectivity and consistency during specificity and performance tests.

Functionalized optic fibers were also chosen by Chiang et al. (2019) [90]. Unlike the method previously reported, this sensor was based on localized surface plasmon resonance (LSPR), which allows an easier and less expensive fabrication. In addition, LSPR is less prone to errors in experimental data due to a smaller decay length which makes this less sensitive to bulk effects and external variables [98,99]. The fiber optic nanogold-linked immunosorbent assay was developed employing an immobilized capture probe on the fiber core surface and a detection probe conjugated to gold nanoparticles in a solution (Figure 5b). The introduction of a sample containing both analyte and detection probe in the microfluidics of the biosensor chip led to the formation of a sandwich-like complex between capture probe-analyte-detection probe on the fiber core surface, which induced the absorption of the fiber optic evanescent wave. This technique provided a fast response, required low-cost instrumentation and showed a lower LOD for PCT when compared to commercial assays for the same analyte.

Tsounidi et al. [97] developed a compact bench-top bioanalytical system for CRP determination in human blood samples, using White Light Reflectance Spectroscopy (WLRS) (Figure 5c). This label-free two-site sandwich immunoassay was able to detect up to 1 ng/mL of the target analyte in 12 min and its dynamic range covered normal values of CRP in plasma and acute inflammation cases. The protein was first bound on a chip immobilized capture antibody and then reacted with the detection antibody. Goat polyclonal antibody (GC019) was used for both capture and detection. WLRS detected the increase in thickness on the silicon chip where the capture antibody was anchored. This fast technique provided accurate bioanalytical results, real time signal monitoring and low cost of consumables and instrumentation. Furthermore, results obtained from analyses of the same samples using standard diagnostic laboratory methods were comparable to those achieved by this system.

Giorgi-Coll et al. [91] recently reported an aptamer-based optical assay for the proof-of concept determination of IL-6 [91]. The optical assay (Figure 5d) was based on the aggregation of gold nanoparticles coated with two complementary “sandwich style” aptamers, each with a different IL-6 target moiety.

Recognition and binding to the complementary aptamer pair from IL-6 caused the aggregation of the functionalized nanoparticles, thus shifting the maximum absorption from red to pink, which could be monitored visually.

As we have mentioned in the previous sections, the diagnostic information would improve if more biomarkers could be detected simultaneously, and optical biosensors have been recently developed in this direction.

Nuria Fabri-Faja et al. developed a phase-sensitive interferometric biosensor with a label-free microarray configuration [92] for the simultaneous and rapid evaluation of different biomarkers such as: proteins (e.g., CRP and IL-6) and miRNAs. The sensor chip was based on lens-free interferometric microscopy and equipped with several metallic nanostructures to allow an efficient immobilization of probes such as antibodies for proteins or oligonucleotides for miRNAs. Proteins could be directly detected with this assay, whereas an additional amplification step was required for miRNAs. Despite its potential, further improvements are required for a clinical application of the device due to the limited dynamic range and LOD.

Lower LOD values were achieved by the Surface Enhanced Raman Scattering (SERS) based detection system developed by Kundu et al. [96]. The use of a AgNPs-laden black phosphorous-based SERS platform, allowed to reach a LOD of 1 pM and 100 fM for IL-3 and PCT, respectively. SERS is a technique based on the enhancement of the Raman scattering due to the presence of metallic nanostructures. In this case, Ag nanoparticles (AgNPs) were grown on black phosphorous (BP) flakes. Their arrangement led to an enhancement factor of 10^14^. The major advantages of this system were the possibility to identify different biomarkers from each other, due to the elevated signal selectivity to molecular structure (fingerprint features of Raman spectroscopy), and the chance of real-time monitoring. Unfortunately, no tests in real matrices were performed. Furthermore, these results were achieved with a bench-top instrument that is more performing than a portable one that is more suitable for point-of-care purposes.

Another interesting SERS-based method proposed by Zhou et al. [94] a sandwich structure AgMNPs/IMs/CPs for the detection of Inflammatory Markers (IMs) such as CRP, IL-6 and PCT, was described. Unlike the previous study, Raman Reporters (RaRs) signals were herein enhanced (not those from the analytes). In particular, 2-mercaptopyridine (2-MPY), 4-nitrophenythiophenol (4-NTP) and 2-naphtiothiol (2-NT) were used as RaRs for CRP, IL-6 and PCT, respectively, since their Raman signals do not overlap with those of other substances present in blood serum. Ag magnetic nanoparticles (AgMNPs) modified with an internal standard (4-mercaptophenylacetonitrile) and a specific aptamer bound the IM; then, core porous shells modified with the aptamer and the RaRs formed the sandwich structure and the bound analytes were separated magnetically from the solution. LODs on the order of fg/mL were achieved for the three targets and the results were consistent with hospital analyses, showing recoveries above 96%. This method allowed the simultaneous, precise, quantitative detection of IMs in serum, providing a rapid screening, accurate evaluation, early monitoring, and diagnosis of sepsis.

#### 3.2.2. Pathogens

Santopolo et al. (2019) [93] developed a new rapid method for identifying urease-producing bacteria based on the detection of urease. The same principle used by Giorgi-Coll et al. for IL-6 detection was exploited for developing this assay: assembled and dispersed functionalized Au nanoparticles exhibit different wavelengths of maximum absorption.

This method substitutes the slow bacteriological culture steps with a 10 min capture procedure. The negatively charged bacteria and proteins are captured on magnetic beads coated with the positively charged polymer poly (diallyldimethylammonium chloride) (PDDA) (Figure 5e). Subsequently, the presence of urease enzymes bound to the beads was detected by adding urea, Au nanoparticles (AuNPs) and bovine serum albumin (BSA), which regulates the colloids aggregation in a manner that depends on pH. In fact, urease-positive bacteria hydrolyze urea to ammonia, increasing the pH and destabilizing the nanoparticles aggregations (red-shifted).

In contrast, urease-negative bacteria do not increase the pH upon the addition of urea, and the BSA triggers the assembly of gold nanoparticles (blue-colored test). This rapid assay can detect pathogens in urine at ultra-low concentrations and requires minimal infrastructure and instrumentation, since colors are easily differentiated by eye. These features make it an ideal solution for the rapid screening of urease-positive bacteria in decentralized healthcare schemes.

A label-free method for pathogen detection in sepsis was proposed by Narayana Iyengar et al. [95]. They developed a colorimetric test for bacteria detection in whole blood, achieving a LOD of 10^3^ CFU/mL in less than 5 h. The assay was divided into two main steps: first, a lysis buffer was added to whole blood and the red blood cells lysis occurs without damaging bacteria; then, the solution was filtered through a 0.45 μm cellulose filter. Viable bacteria, trapped into the filter, were then dispersed in a culture media containing ferric citrate and ferricyanide, incubated at 37 °C and exposed to visible light irradiation. Consequent bacterial proliferation implied the metabolic production of Prussian Blue (PB) molecules that turn the solution from colorless to blue (Figure 5f). The assay results sensitive to both Gram-negative and Gram-positive bacteria and even for mixtures.

## 4. Conclusions

The early detection of sepsis still remains an open challenge in the clinical settings particularly in low-income countries. Sepsis diagnosis comprises the evaluation of the patient’s physiological parameters, the pathogen detection and the biomarkers associated to the host response. Each one of these steps provides useful information necessary to choose the correct treatment and consequently impact the management. Regarding the detection of biomarkers, the acquisition of only one biomarker does not provide useful information to the medical doctor. Indeed, recent studies have reported the difficulties encountered in the selection of a unique biomarker able to univocally represent the sepsis condition. It was stated that in order to obtain more accurate information the diagnosis should be based on the assessment of the levels of different biomarkers such as PCT, CRP and interleukins (e.g., IL-6 and IL-3).

In this context, efforts are being applied to the discovery of new biomarkers. During the diagnostic process, the response time associated to traditional techniques represents a crucial bottleneck, which strongly depends on the time required for sample collection and laboratory analysis procedures. Recent significant advances in the field of biosensors may contribute to reduce the response time for both pathogen and biomarker detection. Different strategies have been developed from the label-free to label-based optical and electrochemical assays, taking advantage of the recent improvements in nanobiotechnology.

Point of care systems able to simultaneously detect different biomarkers represent interesting alternatives as well. From recent works regarding biosensors for sepsis diagnosis presented in this review, it is evident that there is a lack of an integrated point of care system able to detect both pathogens and biomarkers. This gap may be filled in the near future thanks to the rapid and continue advancements in the physics, chemistry, biology, and engineering fields.

## Figures and Tables

**Table 1:** List of main pathogenic bacteria than can cause sepsis [18].

Bacteria	Gram	Respiration
*Staphylococcus aureus*	+	Aerobic/Facultative anaerobic
*Enterococcus faecalis*	+	Aerobic/Facultative anaerobic
*Streptococcus pneumoniae*	+	Aerobic/Facultative anaerobic
*Neisseria meningitidis*	−	Aerobic
*Klebsiella pneumoniae*	−	Aerobic/Facultative anaerobic
*Acinetobacter baumannii*	−	Aerobic
*Escherichia coli*	−	Aerobic/Facultative anaerobic
*Salmonella enterica*	−	Aerobic/Facultative anaerobic
*Shigella dysenteriae*	−	Aerobic/Facultative anaerobic
*Citrobacter freundii*	−	Aerobic/Facultative anaerobic
*Serratia marcescens*	−	Aerobic/Facultative anaerobic
*Proteus mirabilis*	−	Aerobic/Facultative anaerobic
*Pseudomonas aeruginosa*	−	Aerobic/Facultative anaerobic
*Bacteroides fragilis*	−	Obligate anaerobic
*Haemophilus influenzae b*	−	Aerobic/Facultative anaerobic

**Table 2:** List of electrochemical biosensors.

Electrode	Biorecognition Element	Biomarker	Technique	LOD	Working Range	Sample	Response Time	Year	Ref.
Gold	BP3 peptide	PCT	EIS	12.5 ng/mL	0.013–0.25 μg/mL	Buffer	/	2017	[65]
Carbon screen printed	Antibody	PCT	Amperometric	0.1 ng/mL	0.5–1000 ng/mL	Human serum	<20 min	2020	[66]
Gold	Antibody	PCT	Amperometric	0.04 ng/mL	0.1–20 ng/mL	Plasma	<20 min	2020	
Carbon screen printed	Antibody	CRP	Amperometric	0.80 μg/mL	2–100 μg/mL	Plasma	5 min	2020	[67]
Carbon screen printed	Antibody	CRP	Amperometric	0.058 μg/mL	1–100 μg/mL	Plasma	5 min	2021	[68]
Glassy carbon electrode	Antibody	PCT	Amperometric	0.011 pg/mL	0.0001–100 ng/mL	Diluted serum	50 min	2022	[69]
Glassy carbon electrode	Antibody	PCT	DPV	0.46 pg/mL	0.001–100 ng/mL	Diluted human serum	/	2021	[70]
Glassy carbon electrode	Antibody	PCT	DPV	0.3 pg/mL	1 pg/mL–100 ng/mL	Human serum	/	2021	[71]
Gold interdigitated electrode	Antibody	PCT, CRP	EIS	10 ng/mL	0.01–10 ng/mL	Human Serum	<15 min	2019	[72]
Gold electrode	Antibody	PCT, CRP	Amperometric	10 ng/mL	0.01–10 ng/mL	Clinical sample	<15 min	2021	[73]
Gold electrode on microneedle	Antibody	IL-6	DPV	/	20–100 pg/mL	Human Serum spiked	3 min	2018	[74]
Gold interdigitated	Antibody	IL-3	Capacitive	3.0 pg/mL	3.0–100 pg/mL	Human Serum spiked	/	2021	[75]
Gold screen printed	Antibody	IL-3	Chronoamperometry	10 pg/mL	10–10^4^ pg/mL	Plasma/serum from clinical sample	<1 h	2018	[76]
Gold	Antibody	IL-6, IL-8, IL-10, TRAIL, IP 10	EIS	0.1, 0.1, 1.0, 1.0, 1.0 pg/mL	0.01–10^4^, 0.1–5000, 0.1–10^3^, 1.0–2×10^3^ pg/mL	Plasma	5 min	2021	[77]
Disposable sensor cartridge with a gold-based array electrodes	Antibody	IL-6, IL-8, IL-10, TRAIL, IP 10	Label-free non faradic impedence spetroscopy	0.1, 0.1, 1.0, 1.0, 1.0 pg/mL	0.01–10^4^, 0.1–5000, 0.1–10^3^, 1.0–2×10^3^ pg/mL	Clinical samples	5 min	2022	[78]
Gold	RNA specific probe	16S RNA from *S. aureus*, *E coli*, *P aeruginosa*, *P. mirabilis*	Amperometry	290 CFU/mL	/	Human blood	<1 h	2017	[79]
Indium tin oxide coated glass	Conductive MIP	*K. pneumoniae*	DPV	1.35 CFU/mL	1.0–1.0×10^5^ CFU/mL	Spiked human urine	3 min	2022	[80]
Gold	CRISPR/Cas12a	DNA from *E. coli, S. aureus*	EIS	3.0 nM	3 –18 nM	Buffer solution spiked clinical strains	1 h	2022	[81]

Abbreviations: EIS electrochemical impedance spectroscopy, DPV differential pulse voltammetry, MIP molecularly imprinted polymers.

**Table 3:** List of optical biosensors and their main characteristics.

Substrate	Biorecognition Element	Biomarker	Technique	LOD	Working Range	Sample	Response Time	Year	Ref.
Optic fiber	Antibody	CRP	SPR	1.17 μg/mL	0.01–20 ug/mL	PBS buffer	/	2017	[89]
Optic fiber	Antibody	PCT	LSPR	95 fg/mL	1–100 ng/mL	Human serum	<15 min	2019	[90]
AuNPs	Aptamers	IL-6	LSPR	1.95 μg/mL	3.3–125 μg/mL	mixed protein solution	5 min	2020	[91]
gold nanohole array (Au-NHA)	Antibody	CRP	Interferometry	18 mg/mL	0–250 μg/mL	spiked PBS sample	1 min after sample incubation	2019	[92]
	Antibody	IL-6		88 mg/mL	0–400 μg/mL				
	DNA capture probe	miRNA-16		6 mg/mL	0.8–12.5 μg/mL				
AuNPs	electrostatic	urease	LSPR	0.8 μg/mL	0.8–12.5 μg/mL	broth culture	40 min	2019	[93]
AgNPs@BP	Aptamers	CRP	SERS	100 fg/mL	10^−4^–10 ng/mL	Human serum	/	2022	[94]
		IL-6		0.1 fg/mL	10^−7^–10^−2^ ng/mL				
		PCT		1.0 fg/mL	10^−6^–10^−1^ ng/mL				
/	Photocatalysis	*S. capitis*	Colorimetry	10^3^ CFU/mL	10^2^–10^8^ CFU/mL	Whole blood	<5 h	2021	[95]
		*E. coli*							
AgMNPs/CPs	Label-free	IL-3	SERS	1000 fM	1 pM–100 nM	Sterile human serum	Real time	2022	[96]
		PCT		100 fM	100 fM–100 nM				
Silicon chip	Antibody	CRP	WLRS	1 ng/mL	0.05–200 μg/mL	Human plasma	12 min	2021	[97]

Abbreviations: SPR surface plasmon resonance, LSPR localized surface plasmon resonance, AuNPs gold nanoparticles, BP black phosphorous, SERS surface enhanced Raman scattering, AgMNPs silver magnetic nanoparticles, WLRS white light reflectance spectroscopy.

## Data Availability

Not applicable.
